# Green‐Synthesized Silver Nanoparticles Improve Cognitive Function and Modulate Nrf2/HO‐1–BDNF‐Related Gene Expression in Experimental Chronic Toxoplasmosis

**DOI:** 10.1155/jotm/6264261

**Published:** 2026-10-25

**Authors:** Mojtaba Khaksarian, Amal Khudair Khalaf, Saeedeh Shojaee, Pegah Shakib, Amir Javadi Mamaghani, Erfan Babaeinejad, Hossein Mahmoudvand

**Affiliations:** ^1^ Razi Herbal Medicines Research Center Lorestan University of Medical Sciences Khorramabad Iran lums.ac.ir; ^2^ Department of Microbiology College of Medicine University of Thi-Qar Nasiriyah Iraq utq.edu.iq; ^3^ Department of Medical Parasitology Tehran University of Medical Sciences Tehran Iran tums.ac.ir; ^4^ Student Research Committee Lorestan University of Medical Sciences Khorramabad Iran lums.ac.ir; ^5^ Hepatitis Research Center Lorestan University of Medical Sciences Khorramabad Iran lums.ac.ir

**Keywords:** antioxidant, learning, memory, nanomedicine, neurotoxoplasmosis

## Abstract

**Background and Aims:**

This study aimed to evaluate the neuroprotective effects of silver nanoparticles (AgNPs) green‐synthesized using the ethanolic extract of *Muscari neglectum* against *Toxoplasma gondii*‐induced cognitive impairment and to explore the underlying Nrf2/BDNF and oxidative stress–related mechanisms.

**Methods:**

Chronic toxoplasmosis was experimentally induced in mice using the Tehran strain of *T. gondii.* Forty‐eight mice were randomly allocated into six experimental groups, including a noninfected control group, an infected untreated control group, and four treatment groups receiving pyrimethamine (PYM, 20 mg/kg), donepezil (DON, 2 mg/kg), AgNPs (10 mg/kg), or AgNPs (20 mg/kg). Spatial learning and memory capabilities were assessed using the Morris water maze test. Post‐treatment, brain cyst number and size were determined via microscopic examination. Additionally, quantitative real‐time PCR was employed to evaluate the expression levels of genes associated with oxidative stress and neuroprotection, including brain‐derived neurotrophic factor (*Bdnf*), NADPH oxidase 2 (*Nox2*), superoxide dismutase 2 (*Sod2*), catalase (*Cat*), glutathione peroxidase 1 (*Gpx1*), nuclear factor erythroid 2–related factor 2 (*Nrf2*), and heme oxygenase‐1 (*Hmox1*).

**Results:**

Treatment with AgNP resulted in a significant, dose‐dependent improvement in behavioral performance, evidenced by reduced escape latency and enhanced exploration of the target quadrant (*p* < 0.001). Moreover, AgNP administration markedly decreased both the number and size of brain *Toxoplasma* cysts, with the 20 mg/kg dose demonstrating superior efficacy compared to PYM (*p* < 0.01). Molecular analyses indicated significant upregulation of *Nrf2, Hmox1, Bdnf, Sod2, Cat*, and *Gpx1*, accompanied by downregulation of *Nox2* expression in mice treated with AgNP (*p* < 0.001).

**Conclusion:**

These findings suggest that green‐synthesized AgNP exert significant, dose‐dependent antiparasitic and neuroprotective effects in the context of chronic toxoplasmosis. AgNP notably reduced brain cyst burden, enhanced spatial learning and memory, and normalized oxidative stress‐related gene expression. Importantly, AgNP exhibited greater efficacy than both PYM and DON, indicating their potential as a promising therapeutic approach for neurotoxoplasmosis.

## 1. Introduction

*Toxoplasma gondii* is a protozoan parasite with a global distribution, capable of infecting nearly all warm‐blooded animals, including humans [1]. Felids serve as the definitive hosts, releasing environmentally resilient oocysts that contaminate soil, water, and food sources. Human infection primarily occurs through the ingestion of tissue cysts present in undercooked meat, consumption of food or water contaminated with oocysts, and vertical transmission from mother to fetus, which can lead to severe congenital disease [2]. Epidemiological data indicate that toxoplasmosis affects approximately one‐third of the world’s population, with seroprevalence rates strongly influenced by dietary practices, sanitation standards, and geographic location [3].

Beyond its classical infectious characteristics, accumulating evidence suggests that chronic *T. gondii* infection significantly impacts the central nervous system, particularly affecting hippocampal‐dependent cognitive functions [4]. Experimental investigations using rodent models have consistently revealed marked impairments in learning and memory, including deficits in spatial navigation, recognition memory, and associative fear conditioning [5, 6]. These cognitive alterations correspond to dysfunction within hippocampal and amygdalar neural circuits and are mechanistically linked to compromised synaptic plasticity, diminished long‐term potentiation (LTP), and disrupted processes of memory consolidation and retrieval [6]. Notably, these cognitive impairments are underpinned by neurobiological changes, such as persistent microglial activation, chronic low‐grade neuroinflammation, and elevated production of reactive oxygen species (ROS) [6, 7]. In this context, increased generation of ROS via NADPH oxidase 2 (NOX2), coupled with inadequate antioxidant defenses mediated by enzymes including superoxide dismutase 2 (SOD2), catalase (CAT), and glutathione peroxidase 1 (GPX1), establishes a sustained oxidative milieu that inflicts direct damage on synaptic proteins and neuronal membranes within the hippocampus. Concurrently, dysregulation of the nuclear factor erythroid 2–related factor 2 (NRF2) signaling pathway and its downstream effector heme oxygenase‐1 (HMOX1) further compromises endogenous antioxidant responses, thereby exacerbating oxidative injury [8–10]. Oxidative stress and neuroinflammation critically intersect within neurotrophic signaling pathways, notably those involving brain‐derived neurotrophic factor (BDNF), which plays a pivotal role in synaptic plasticity, dendritic remodeling, and memory consolidation [11]. Diminished BDNF expression and disrupted tropomyosin receptor kinase B (TrkB) signaling have been linked to deficits in learning and memory observed in chronic neuroinflammatory disorders, including toxoplasmosis [12].

The current standard treatment regimen for toxoplasmosis involves the administration of pyrimethamine (PYM) combined with sulfadiazine, agents that inhibit parasite folate metabolism and are primarily effective against the rapidly proliferating tachyzoite stage [13]. Nevertheless, these pharmacological interventions exhibit limited efficacy against the latent bradyzoite cysts residing within the central nervous system and are frequently associated with adverse hematological and gastrointestinal effects [14]. Consequently, chronic infection often persists despite therapeutic intervention, and the resultant cognitive and behavioral impairments may remain partially or entirely unresolved. These constraints underscore the critical necessity for the development of alternative therapeutic approaches that focus on host‐directed mechanisms, specifically the modulation of oxidative stress and the restoration of neurotrophic factors, in order to alleviate neurocognitive impairments resulting from infection.

Nanotechnology involves the design and manufacturing of materials and systems at the nanometer scale, which exhibit unique biological and pharmacological characteristics in contrast to their traditional counterparts [15]. The green synthesis methodology, which employs plant extracts as both reducing and capping agents, offers an environmentally sustainable and biocompatible alternative to traditional chemical and physical synthesis techniques. Phytochemicals such as flavonoids, phenolics, and terpenoids not only facilitate the formation of nanoparticles but also augment their biological activity while mitigating systemic toxicity [16]. Green‐synthesized metal nanoparticles, with a particular emphasis on silver nanoparticles (AgNPs), have garnered considerable attention as potential biomedical agents owing to their distinctive physicochemical characteristics, elevated surface reactivity, and extensive antimicrobial efficacy [17]. In the realm of parasitic infections, AgNP has exhibited notable antiparasitic effects against various protozoan pathogens, including *T. gondii, Leishmania* spp., *Giardia* spp*.,* and *Ascaris* spp. [18]. The proposed mechanisms underlying these effects encompass disruption of parasite membrane integrity, induction of excessive ROS, mitochondrial dysfunction, and inhibition of critical metabolic enzymes, culminating in parasite death. Moreover, AgNP may influence host immune responses by attenuating proinflammatory cytokine production and reducing parasite‐induced tissue damage [18]. These attributes render AgNP promising candidates for therapeutic intervention in both acute and chronic phases of toxoplasmosis, particularly within the central nervous system.

Beyond their antiparasitic properties, accumulating evidence indicates that biosynthesized AgNP possess neuroprotective capabilities [19]. These effects are primarily mediated through the attenuation of oxidative stress, suppression of neuroinflammatory pathways, and enhancement of endogenous antioxidant defense systems, including SOD, CAT, and GPX [20]. Notably, the nanoparticle‐induced reduction of oxidative stress is closely linked to the preservation of synaptic integrity and improved gene expression patterns consistent with neuroprotective signaling in hippocampal regions, which are essential for cognitive functions such as learning and memory [20]. Furthermore, emerging research suggests that modulation of neurotrophic signaling pathways, particularly involving BDNF, may underlie the cognitive improvements associated with nanoparticle treatment [11]. By restoring redox homeostasis and potential modulation of neuroinflammatory pathways, pending further validation, green‐synthesized nanoparticles may indirectly promote BDNF expression and synaptic plasticity, thereby enhancing cognitive processes including learning, memory consolidation, and behavioral performance. We hypothesized that in parasitic disease models such as toxoplasmosis, where chronic infection is linked to oxidative stress, neuroinflammation, and diminished BDNF signaling, AgNP represent a promising therapeutic approach to ameliorate neurocognitive impairments through a multifaceted mechanism targeting the oxidative stress–inflammation–BDNF axis. While previous studies have investigated the effects of AgNPs against *T. gondii* infection, the present study is, to the best of our knowledge, the first to specifically examine the neuroprotective potential of AgNPs synthesized from *Muscari neglectum* extract in a chronic toxoplasmosis model, with a focus on cognitive impairment and Nrf2/BDNF‐related signaling pathways.

## 2. Materials and Methods

### 2.1. Chemicals

Silver nitrate (AgNO_3_, purity ≥ 99.9%, Cat No. 7761‐88‐8), donepezil (DON, purity ≥ 98%, Cat No. 120011‐70‐3), and PYM (Cat No. 58‐14‐0) were purchased from Sigma‐Aldrich, Germany. DON was included as a reference neuroprotective agent due to its established efficacy in improving learning and memory deficits. While DON’s primary pharmacological action is cholinergic (acetylcholinesterase inhibition), its inclusion was intended to provide a symptomatic cognitive benchmark rather than a mechanistic comparator for the Nrf2/HO‐1 pathway. Therefore, comparisons between AgNP and DON should be interpreted in the context of their distinct mechanisms of action. PYM (20 mg/kg) was used as a reference anti‐*Toxoplasma* agent due to its established efficacy as a first‐line therapy against *T. gondii* infection.

### 2.2. Green Synthesis of AgNP

#### 2.2.1. Preparation of Ethanolic Extract

*M. neglectum* was specifically selected for AgNP synthesis due to its well‐documented phytochemical profile, particularly its richness in flavonoids, phenolic acids, and homoisoflavonoids, which are known to act as effective reducing and capping agents during nanoparticle biosynthesis. Moreover, the antioxidant and anti‐inflammatory properties attributed to this plant are mechanistically consistent with the neuroprotective objectives of the present study, providing dual biological relevance to both the synthesis process and the therapeutic application. A 50 g sample of dried flowers of *M*. *neglectum* was collected during the spring of 2025 from rural areas within the Bastam district of Aleshtar city, located in Western Iran. The plant material was taxonomically authenticated by Dr. Javad Ghasemian Yadegari, a botanist at the Department of Pharmacognosy, Razi Herbal Medicines Research Center, Khorramabad, Iran. A voucher specimen (No. 140372) has been deposited in the Herbarium of the Razi Herbal Medicines Research Center for future reference. The collection was performed on privately owned land, and no special permissions were required. Additionally, the study adhered to ethical guidelines, ensuring that no endangered or protected species were involved, in accordance with the IUCN Policy Statement on Research Involving Species at Risk of Extinction and the Convention on International Trade in Endangered Species of Wild Fauna and Flora (CITES). The plant material was ground into a fine powder and subjected to extraction by soaking in 80% methanol for 48 h. The resulting extract was filtered and concentrated under reduced pressure using a rotary evaporator [21].

#### 2.2.2. Green Synthesis Procedure

AgNP were synthesized by combining 5 mL of the plant extract with 95 mL of a 1 mM AgNO_3_ solution, followed by continuous stirring at 60°C for 2 h at an approximately neutral pH (∼7.2). The formation of AgNP was indicated by a color change to brown. The precipitate was subsequently washed three times with distilled water, each wash followed by centrifugation at 13,000 rpm for 20 min. A final wash with ethanol was performed, and the nanoparticles were dried by heating at 75°C in an oven. The synthesis yield, calculated based on dry weight recovery, was approximately 13.2%. The entire synthesis procedure was independently replicated three times, producing consistent nanoparticle characteristics and thereby confirming the reproducibility of the method [22].

#### 2.2.3. UV–Visible Spectroscopy for Nanoparticle Confirmation

The synthesis of AgNP was initially confirmed by the observable color change and further validated through UV–visible absorption spectroscopy. Absorption spectra of the synthesized nanoparticles were recorded and compared with those of the plant extract over the wavelength range of 200–700 nm. The presence of characteristic absorption peaks corresponding to AgNP confirmed successful nanoparticle formation.

#### 2.2.4. Scanning Electron Microscopy (SEM) and Dynamic Light Scattering (DLS) Analysis

The morphological characteristics and surface topology of the synthesized AgNP were examined using a Hitachi S‐4160 scanning electron microscope operating at an accelerating voltage of 15 kV, with magnifications up to 10,000×, enabling nanometer‐scale resolution. Particle size and distribution within the colloidal suspension were evaluated by DLS.

#### 2.2.5. Fourier‐Transform Infrared (FTIR) Spectroscopy

To elucidate the functional groups involved in nanoparticle synthesis and stabilization, 25 mg of dried AgNP was thoroughly mixed with 250 mg of potassium bromide (KBr) and compressed into a pellet. FTIR spectra were recorded using an Agilent Cary 630 spectrometer (Waldbronn, Germany) over the spectral range of 400–4000 cm^−1^. The resulting spectra provided insights into the surface chemistry and capping agents associated with the nanoparticles.

### 2.3. Animals and Housing Conditions

Male BALB/c mice (*n* = 48), approximately six weeks old and weighing between 25 and 30 g, were obtained from the accredited breeding facility at Lorestan University of Medical Sciences, Iran. Throughout the duration of the study, the animals were housed under controlled laboratory conditions, maintained at a temperature of 24°C ± 1, with relative humidity ranging from 40% to 70%, and with a 12‐h light/dark cycle. The mice were provided with *ad libitum* access to a standard pelleted diet and drinking water. Animals were randomly assigned to experimental groups using a computer‐generated randomization sequence. Investigators involved in outcome assessment were blinded to group allocation to minimize potential bias. The sample size (*n* = 8 per group) was selected based on previous comparable in vivo studies and preliminary data indicating adequate statistical power to detect biologically relevant differences. BALB/c mice were selected as the experimental model based on their well‐established susceptibility to chronic *T. gondii* infection. Their predominantly Th2‐biased immune profile facilitates bradyzoite cyst formation and persistence in brain tissue without causing acute lethality, rendering them the most widely validated murine strain for chronic toxoplasmosis models [22]. This strain also offers consistency with the majority of published literature in the field, ensuring comparability of the present findings.

### 2.4. Ethical Statement

All experimental procedures were conducted in accordance with internationally accepted guidelines for the care and use of laboratory animals (ARRIVE guidelines and NIH recommendations) and were approved by the Institutional Animal Ethics Committee of Lorestan University of Medical Sciences, Khorramabad, Iran (Approval No: IR.LUMS.REC.1398.021).

### 2.5. Induction of Infection and Establishment of Chronic Toxoplasmosis

A chronic toxoplasmosis model was established using the Tehran strain of *T. gondii*. Each mouse received an intraperitoneal injection of 0.5 mL of brain homogenate obtained from an infected donor mouse and containing approximately 20–25 tissue cysts. Although the oral route more closely reflects the natural acquisition of toxoplasmosis, the intraperitoneal administration of a defined cyst‐containing inoculum was selected to establish a reproducible and synchronized chronic infection with consistent brain cyst formation across experimental animals [23].

### 2.6. Study Design and Grouping

The experimental mice were randomly divided into six separate groups, each consisting of eight individuals, including:

 I. Noninfected control
 II. Infected untreated control
 III. Infected + PYM
 IV. Infected + DON
 V. Infected + AgNPs 10 mg/kg
 VI. Infected + AgNPs 20 mg/kg

The study included noninfected and infected untreated controls, together with PYM and DON reference‐treatment groups. These controls enabled assessment of infection‐associated changes and comparison of AgNP treatment with established antiparasitic and cognitive reference treatments. Nevertheless, pathway‐specific intervention and protein‐level validation were not included and would be necessary to establish a causal mechanistic relationship.

### 2.7. Dosing Formulation and Oral Administration

The dosages of AgNP (10 and 20 mg/kg) were selected based on prior literature demonstrating that these concentrations are both nontoxic and pharmacologically effective in murine models [21, 22] and were corroborated by preliminary pilot studies. All compounds were solubilized in normal saline, with saline‐treated animals serving as vehicle controls. Therapeutic interventions in the infected groups commenced six weeks after infection and continued for 14 consecutive days. Throughout the study period, survival rates and body weights were monitored daily. The primary outcome measure was the reduction in parasite burden, while secondary outcomes included cytokine profiling, assessment of oxidative stress biomarkers, and gene expression analyses. All evaluations were conducted by investigators blinded to group assignments to reduce potential bias. All orally administered treatments were delivered at a dosing volume of 10 mL/kg. The suspensions were freshly prepared and sonicated for 10 min immediately before administration to ensure homogeneity and minimize aggregation. All other treatments were administered according to their respective dosing protocols.

### 2.8. Behavioral Assessment

#### 2.8.1. Morris Water Maze (MWM) Test

The MWM task was employed to assess spatial learning and memory capabilities [19]. The apparatus consisted of a black circular pool, 160 cm in diameter and 80 cm in height, coated with nontoxic black paint. The pool was filled with water maintained at room temperature to a depth of 40 cm. The pool surface was divided into four equal quadrants, with designated starting points labeled as North (N), South (S), East (E), and West (W). A square platform, measuring 10 cm in diameter, was submerged 1.5 cm below the water surface and positioned centrally within the northeast quadrant. The experiments were conducted in a dimly lit room, where fixed geometric cues (e.g., circles, squares, and triangles) were affixed at various locations on the surrounding walls to serve as extra‐maze spatial references. Animal movements were recorded using a computerized video tracking system (Noldus Ethovision® system, Version 5, USA), allowing real‐time monitoring on a computer screen [24].

#### 2.8.2. Spatial Learning

Spatial learning trials were initiated at Week 8 postinfection, following confirmation of chronic toxoplasmosis by microscopic assessment of brain tissue cysts. During the spatial acquisition phase, mice were subjected to four training trials per block, each trial lasting up to 60 s, with an intertrial interval of 60 s. These trials were repeated across three blocks, separated by 30‐min interblock intervals. Upon locating the submerged platform, animals were permitted to rest on it for 20 to 30 s. Subsequently, mice were gently dried with a towel and returned to their home cages. After a rest period of 20 to 30 s, they were reintroduced into the maze for the subsequent trial. If a mouse failed to find the platform within the allotted 60 s, the experimenter guided it onto the platform. For each trial, mice were released randomly from one of the four quadrants, facing the pool wall at the release point. Each subject experienced all four release locations across trials. Key performance metrics, including latency to reach the platform and the distance traveled, were recorded for each trial.

#### 2.8.3. Short‐Term Spatial Memory

The 2‐h inter‐trial interval between the final acquisition trial and the probe test was selected based on well‐established MWM protocols for evaluating short‐term hippocampal‐dependent spatial memory retention in rodent models, as previously described [25]. During this test, the platform was removed, and each mouse was allowed to swim freely for 60 s. The duration and distance spent within the target quadrant (Quadrant 4) were quantified as indicators of memory retention [25].

#### 2.8.4. Latency to Visible Platform and Swimming Speed

Subsequent to the probe test, a visible platform trial was conducted to assess potential effects of *Toxoplasma* infection and treatments on sensory‐motor coordination and motivation. In this assessment, the platform was elevated 2 cm above the water surface and made conspicuous by covering it with aluminum foil. The animals’ ability to locate and escape onto the visible platform was measured to control for noncognitive factors influencing performance [25].

### 2.9. Brain Tissue Collection

All mice were administered deep anesthesia through an intraperitoneal injection of a ketamine and xylazine mixture at a dosage ratio of 100 mg/kg to 10 mg/kg, respectively [26]. Under aseptic conditions, whole brain tissues were harvested from mice and transferred into sterile microtubes. Samples were subsequently stored at −80°C until molecular testing.

### 2.10. Parasitological Assessment

Brain tissue smears were prepared from standardized portions of brain homogenate, and tissue cysts were counted microscopically under (40×). Cyst burden was expressed as the number of cysts observed per mL of brain homogenate/standardized brain tissue sample. Cyst diameter was measured using a calibrated ocular micrometer [27].

### 2.11. Gene Expression Analysis by Real‐Time PCR

#### 2.11.1. RNA Extraction

Total RNA was extracted from the brain tissue following the manufacturer’s protocol provided with the commercial extraction kit (Yekta Tajhiz Azma, Iran).

#### 2.11.2. RNA Quantification and Quality Assessment

The concentration and purity of the isolated RNA were measured using a Nanodrop spectrophotometer at an absorbance ratio of 260/280 nm. The quality and purity of the extracted RNA were evaluated prior to enzymatic treatment. To prevent amplification artifacts caused by residual genomic DNA contamination, RNA samples were treated with RNase‐free DNase I (Fermentas, USA) before proceeding to complementary DNA (cDNA) synthesis.

#### 2.11.3. cDNA Synthesis

The extracted RNA was reverse transcribed into cDNA using a commercial kit from Yekta Tajhiz Azma, Iran, thereby stabilizing the nucleic acid for subsequent PCR amplification.

#### 2.11.4. Primers

Primers targeting *Nrf2, Hmox1, Nox2, Bdnf, Sod2, Cat, Gpx1,* and *β-actin* genes were designed based on sequences obtained from the NCBI database and validated for specificity using Primer‐BLAST software (Table 1). *β-Actin* served as the internal control gene (housekeeping gene) for normalization of gene expression data. Primer amplification efficiencies ranged between 93% and 105%, with correlation coefficients (*R* ^2^) exceeding 0.99 for all assays.

**TABLE 1:** The sequence of the primers used for evaluating the expression level.

Target genes	Primer sequences (5′‐3′)	Amplicon sizes (bp)
*Bdnf*	F: AACCTGCTGGTGTGTGACGTTC R: CAGCACGAGGCTTTTTTGTTGT	125

*Sof2*	F: GACCCATTGCAAGGAACAA R: GTAGTAAGCGTGCTCCCACAC	120

*Cat*	F: AGCCTGCGTCCTGAGTCTCTG R: ATCCATATCCGTTCATGTGCCTGTG	115

*Gpx1*	F: GAAGTGTGAGGTGAATGGCG R: CTCGAAGTTCCATGCGATGT	100

*Nox2*	F: CGAAGACAACTGGAAGGAA R: GCTCCCACTAACATCACCAC	140

*Nrf2*	F: GCCCAGTCTTCATTGCTCCT R: AGCTCCTCCCAAACTTGCTC	110

*Hmox1*	F: AACTTTCAGAAGGGCCAGGT R: GGGTTCTCCTTGTTGTGCTC	120

*β-Actin*	F: GTGACGTTGACATCCGTAAAGA R: GCCGGACTCATCGTACTCC	124

#### 2.11.5. Real‐Time PCR Amplification

Real‐time PCR was conducted utilizing a SYBR Green‐based Master Mix (Qiagen Rotor‐Gene Q system). Each 20 μL reaction mixture comprised the following components: 10 μL of 2X Master Mix, 0.5 μL each of forward and reverse primers, 1 μL of cDNA template, and 8 μL of sterile distilled water. All reactions were performed in duplicate. The thermal cycling conditions were as follows: initial denaturation at 95°C for 3 min; 40 cycles of denaturation at 95°C for 30 s; and annealing at 60°C for 45 s. Expression levels were quantified employing the optical system software (iQTM5 model, Bio‐Rad, Hercules, CA) by applying the 2^−ΔΔCT^ method. Melt curve analysis was performed following amplification to confirm primer specificity and the absence of nonspecific amplification products. Relative gene expression levels were normalized to β‐actin and calculated relative to the healthy noninfected control group using the 2^−ΔΔCt^ method. In all gene expression assays, each sample represents an independent biological replicate (individual animal, *n* = 8 per group). All real‐time PCR reactions were performed in technical duplicate for each biological replicate. Results are reported as mean ± SEM of biological replicates.

### 2.12. Statistical Analysis

Data analysis for biochemical assays, behavioral evaluations, and molecular studies was performed using SPSS software. Results are presented as mean ± standard error of the mean (mean ± SEM). Learning trial data from the MWM behavioral test were analyzed using repeated measures analysis of variance (R‐ANOVA). One‐way ANOVA was applied to assess memory performance in both the MWM and shuttle box tests, as well as to examine biochemical parameters and gene expression levels. Furthermore, paired *t*‐tests were conducted to evaluate within‐group differences in the shuttle box test. Upon detection of significant effects, post hoc analyses were carried out using Games–Howell and Tukey tests to identify specific group differences. Statistical significance was defined at *p* < 0.05.

## 3. Results

### 3.1. Physicochemical Characterization of AgNP

#### 3.1.1. UV–Visible Spectroscopy Analysis

During the synthesis of AgNPs, a notable color transition of the solution to black was observed, indicating the successful formation of AgNP. The UV–visible absorption spectrum of the green‐synthesized AgNP, prepared using the ethanolic extract of *M. neglectum*, exhibited a distinct SPR peak within the 400–450 nm wavelength range. This SPR peak serves as confirmation of nanoparticle formation. However, detailed information regarding particle morphology and size distribution was further elucidated through SEM and DLS analyses. In contrast, the UV–vis spectrum of the plant extract alone showed absorption peaks predominantly in the ultraviolet region (200–300 nm), which are attributed to the presence of phenolic compounds, flavonoids, and other bioactive constituents inherent to the extract (Figure 1).

**FIGURE 1:**
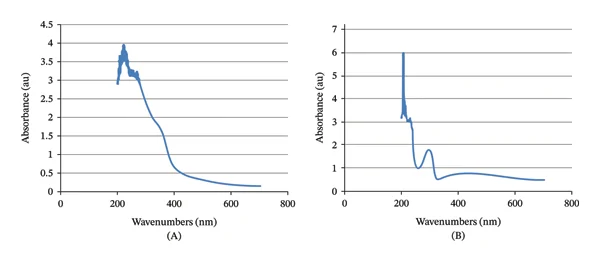
UV–vis spectrometry analysis of (A) ethanolic extract of *Muscari neglectum* and (B) silver nanoparticles (AgNPs) synthesized.

#### 3.1.2. SEM and DLS Analysis

SEM imaging revealed that the synthesized nanoparticles exhibited irregular morphologies and predominantly formed aggregated clusters. The particle distribution was heterogeneous, with evident clustering at the nanometer scale. At a magnification of 30,000× and a scale bar of 1 μm, the particle sizes were estimated to range from approximately 30 to 50 nm. DLS analysis revealed a hydrodynamic particle size distribution of approximately 50–100 nm, with a polydispersity index below 0.3, indicating a relatively narrow size distribution. A minor secondary peak detected within the 1000–2000 nm range was attributed to transient nanoparticle aggregation in the aqueous medium (Figure 2).

**FIGURE 2:**
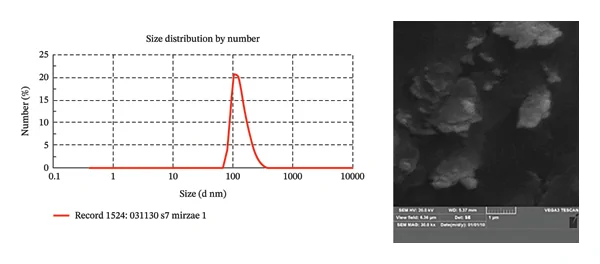
Dynamic light scattering (left) and scanning electron microscope (right) characterization of green‐synthesized silver nanoparticles.

#### 3.1.3. FTIR Spectroscopy

The FTIR spectrum of the ethanolic extract of *M. neglectum* displayed a broad absorption band centered around 3400 cm^−1^, corresponding to O–H stretching vibrations associated with phenolic compounds and alcohol functional groups. A weak absorption band at 2924 cm^−1^ was assigned to C–H stretching vibrations characteristic of aliphatic compounds. A prominent peak observed at 1635 cm^−1^ was attributed to C=O stretching vibrations of carbonyl groups, likely originating from flavonoids or carboxylic acids. Furthermore, absorption bands within the 1400–1000 cm^−1^ range were ascribed to C–O stretching vibrations of polysaccharides or C–N bonds present in proteins (Figure 3). Comparative FTIR analysis between the crude ethanolic extract and the synthesized AgNPs revealed notable spectral differences. A broadening and slight red‐shift of the O–H stretching vibration (∼3400 cm^−1^) in the AgNP spectrum indicated surface adsorption of hydroxyl‐bearing phytochemicals onto the nanoparticle surface. A decrease in C=O peak intensity (∼1635 cm^−1^) suggested involvement of phenolic carbonyl groups in the bioreduction of Ag^+^ ions. Additionally, shifts in absorption bands in the 1400–1650 cm^−1^ region implied conformational changes upon phytochemical binding to nanoparticle surfaces. These differences collectively confirm successful surface functionalization of AgNPs with bioactive constituents of *M. neglectum* extract, consistent with phytochemical‐mediated capping and stabilization.

**FIGURE 3:**
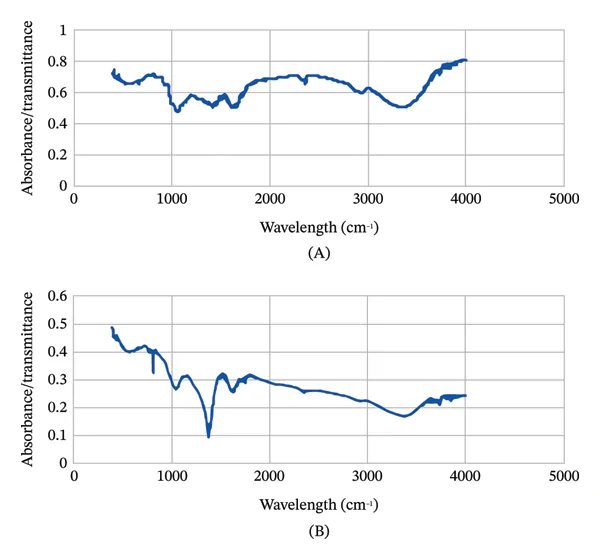
Fourier‐transform infrared spectroscopy (FTIR) analysis of (A) ethanolic extract of *Muscari neglectum* and (B) silver nanoparticles (AgNPs) synthesized.

### 3.2. Behavioral Assessments

#### 3.2.1. Spatial Learning

Analysis of the data derived from the spatial learning performance of animals in the study groups using the MWM revealed that both the distance traveled and the time required to locate the hidden platform were significantly greater in the infected untreated control group compared to the control groups (*p* < 0.05). These findings suggest the presence of spatial learning impairments in the animals affected by toxoplasmosis (Figure 4A,B). Administration of AgNP markedly decreased both the distance traveled (AgNP 10 mg/kg: 741.6 cm and AgNP 20 mg/kg: 618.2 cm) and the escape latency (AgNP 10 mg/kg: 45.3 s and AgNP 20 mg/kg: 34.1 s) to reach the platform in mice infected with *T. gondii*, compared to untreated infected controls. Notably, the 20 mg/kg dosage produced a significantly greater reduction than the 10 mg/kg dosage (*p* < 0.001) as well as all other treatment groups (*p* < 0.01). Furthermore, the ANOVA analysis revealed no statistically significant differences in swimming speed across all experimental groups (Figure 4C).

**FIGURE 4:**
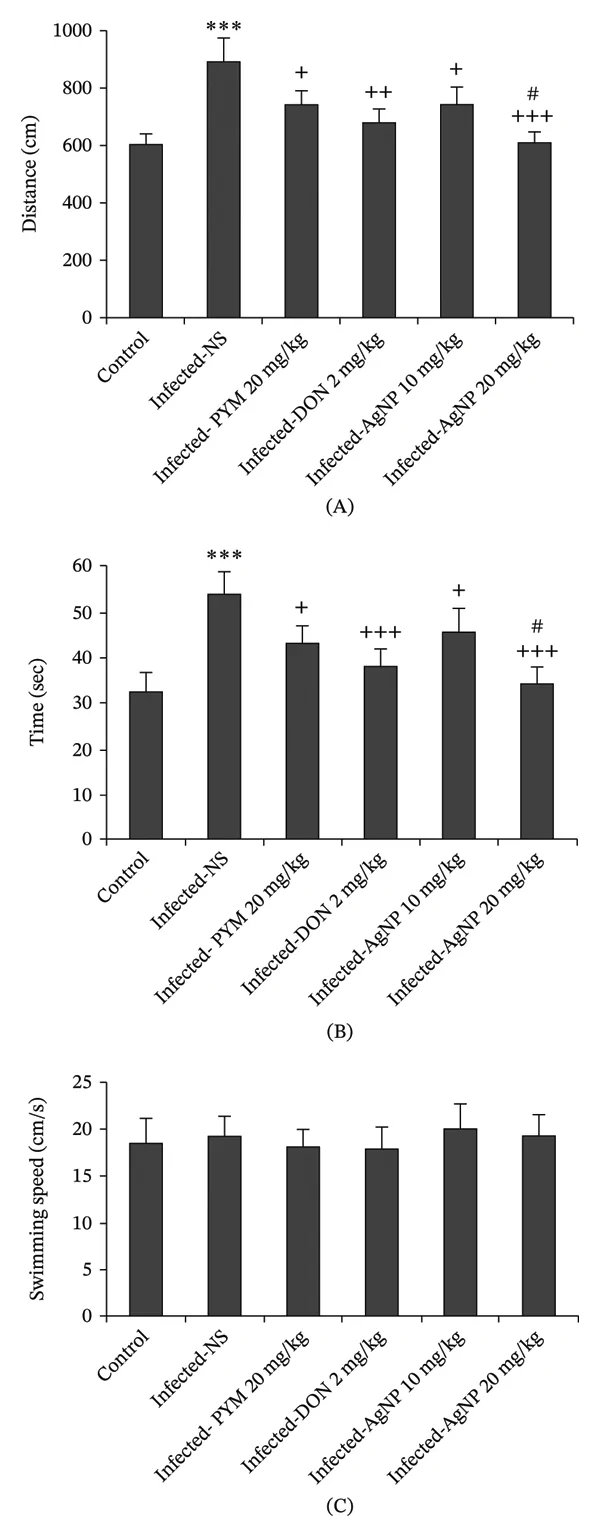
The impaired learning performance observed in the *Toxoplasma*‐infected group compared to the control groups during the Morris water maze task. This impairment was assessed by measuring the distance traveled (A) and the time taken (B) to locate the hidden platform. Following infection, subjects were treated with green‐synthesized silver nanoparticles (AgNP), donepezil (DON), and pyrimethamine (PYM). No significant differences in swimming speed were observed among all groups (C) (*n* = 8 per group). Statistical significance is indicated as follows: ^∗∗∗^ *p* < 0.001 relative to the control group; ^+^ *p* < 0.05, ^++^ *p* < 0.01, and ^+++^ *p* < 0.001 relative to the *T. gondii*‐infected group; and ^#^ *p* < 0.05 when compared to the infected group treated with DON. Post hoc comparisons were performed using Tukey’s honest significant difference (HSD) test following significant one‐way ANOVA results.

#### 3.2.2. Short‐Term Spatial Memory

The results demonstrated that mice infected with *Toxoplasma* exhibited a significant reduction in both the distance traveled and the duration spent (*p* < 0.001) within the target quadrant relative to the control groups (Figure 5A,B). These findings suggest the presence of short‐term memory deficits in the *T. gondii*‐infected mice. The results obtained demonstrated that treatment with AgNP mitigated the effects of *T. gondii* infection. Specifically, infected mice administered AgNP exhibited significantly increased distance traveled (AgNP 10 mg/kg: 26.3 m and AgNP 20 mg/kg: 31.4 m) and time spent (AgNP 10 mg/kg: 25.6 s and AgNP 20 mg/kg: 29.8 s) in the target quadrant relative to the untreated infected group (*p* < 0.001). Importantly, the 20 mg/kg dosage elicited a markedly greater improvement compared to the 10 mg/kg dosage (*p* < 0.001) and all other treatment groups (*p* < 0.01). Furthermore, ANOVA analysis revealed a significant difference in the number of crossings (AgNP 10 mg/kg: 3.26 and AgNP 20 mg/kg: 4.69) among the *T. gondii*‐infected groups compared to the control group (*p* < 0.001; Figure 2C). Infected mice treated with AgNP showed a significant increase in crossing number relative to untreated infected mice (*p* < 0.001). Notably, the 20 mg/kg dosage resulted in a significantly greater reduction than the 10 mg/kg dosage (*p* < 0.01) and treatment groups PYM (*p* < 0.01) and DON (*p* < 0.05).

**FIGURE 5:**
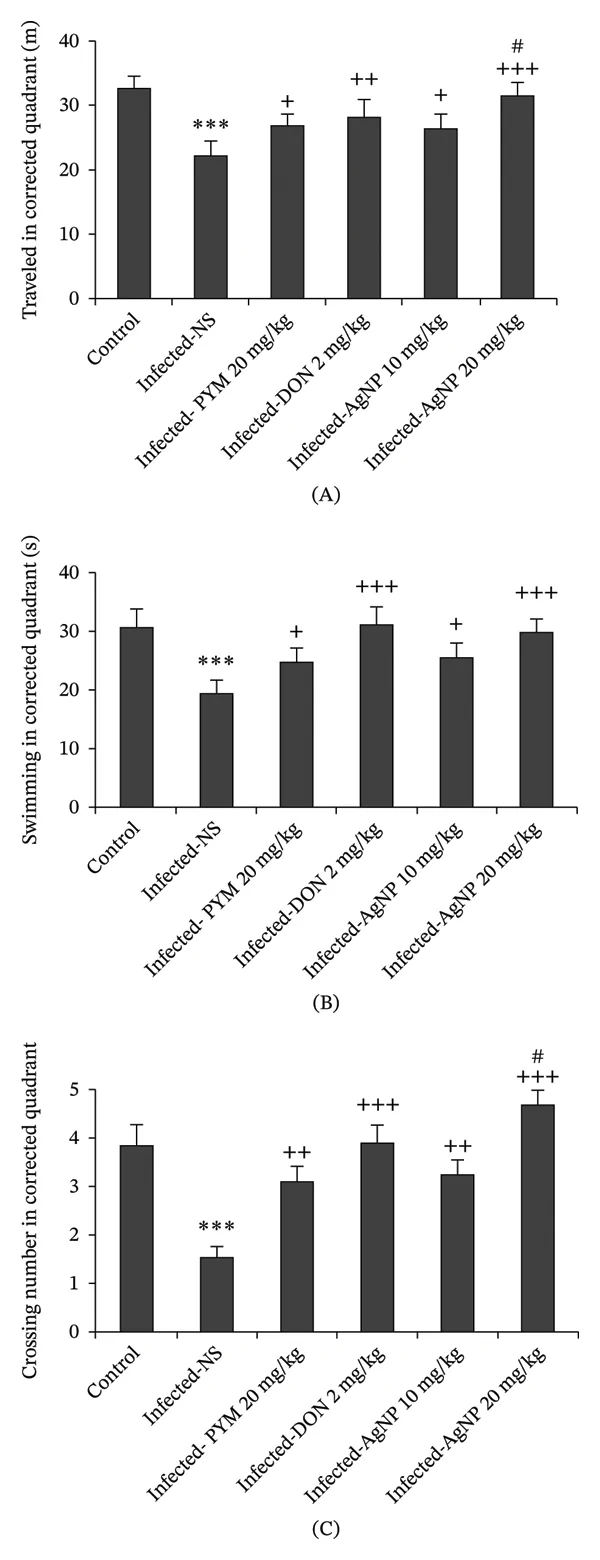
The impact of green‐synthesized silver nanoparticles (AgNP), donepezil (DON), and pyrimethamine (PYM) on spatial short‐term memory impairments induced by *Toxoplasma gondii* infection in mice by measuring the distance traveled (A) and the time spent (B) within the target quadrant and the frequency of crossings (C) over the platform area (*n* = 8 per group). Statistical significance is indicated as follows: ^∗∗∗^ *p* < 0.001 relative to the control group; ^+^ *p* < 0.05, ^++^ *p* < 0.01, and ^+++^ *p* < 0.001 relative to the *T. gondii*‐infected group; and ^#^ *p* < 0.05 when compared to the infected group treated with DON. Statistical significance is indicated as follows: ^∗^ *p* < 0.05 relative to the control group; ^+^ *p* < 0.05, ^++^ *p* < 0.01, and ^+++^ *p* < 0.001 relative to the *T. gondii*‐infected group; and ^#^ *p* < 0.05 when compared to the infected group treated with DON. Post hoc comparisons were performed using Tukey’s honest significant difference (HSD) test following significant one‐way ANOVA results.

#### 3.2.3. Latency to Reach the Visible Platform and Swimming Velocity

As presented in Figure 6, mice across all experimental groups exhibited comparable escape latencies and swimming speeds in the MWM test, indicating the absence of significant differences in visual acuity and motor function among the groups.

**FIGURE 6:**
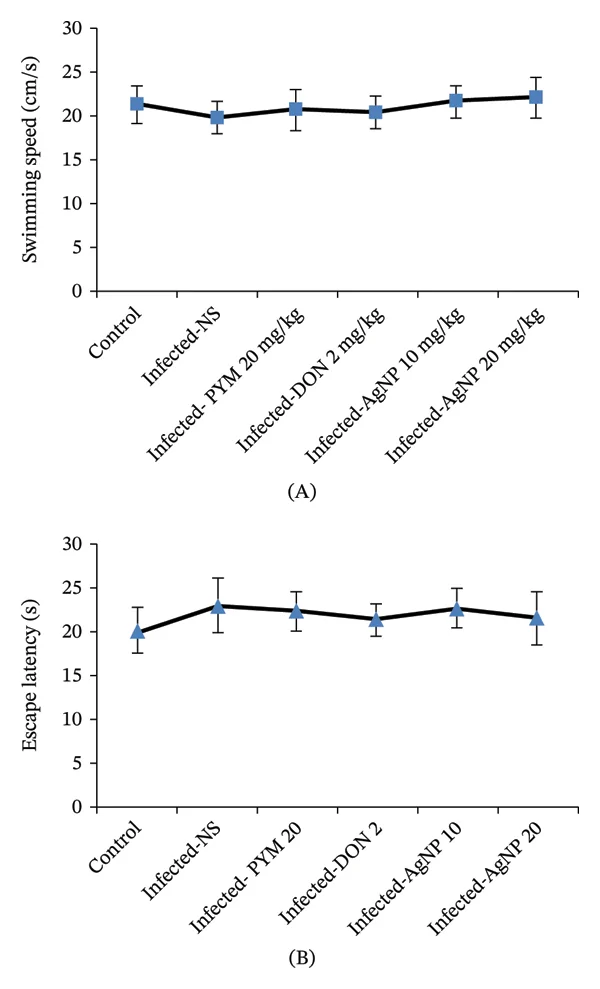
Comparative analyses of swimming velocity (A) and escape latency (B) to the visible platform in the Morris water maze across experimental groups (green‐synthesized silver nanoparticles [AgNP], donepezil [DON], and pyrimethamine [PYM], revealing no statistically significant differences). Data are presented as means ± standard error of the mean (SEM) with a sample size of eight mice per group. Post hoc comparisons were performed using Tukey’s honest significant difference (HSD) test following significant one‐way ANOVA results.

### 3.3. Parasitological Assessment

Chronic *T. gondii* infection in the untreated group (infected‐NS) resulted in a high parasite burden, as evidenced by a mean cyst number of 367.5 and a mean cyst size of 102 μm, confirming successful establishment of chronic infection. Treatment with green‐synthesized AgNP demonstrated a dose‐dependent antiparasitic effect. At 10 mg/kg, AgNP significantly reduced cyst number to 71.2 (∼80.6% reduction, *p* < 0.001). The higher dose (20 mg/kg) reduced cyst number to 21.2 (∼94.2% reduction, *p* < 0.001). Treatment with green‐synthesized AgNPs significantly reduced both brain cyst number and cyst diameter in a dose‐dependent manner. Notably, the 20 mg/kg AgNP dose produced significantly greater reductions in cyst number and size than both the infected untreated control and PYM‐treated groups (*p* < 0.01–0.001). Comparative analysis revealed that AgNP (20 mg/kg) was significantly more effective than PYM in reducing both cyst burden and size (*p* < 0.05–0.01), while AgNP (10 mg/kg) exhibited a strong but slightly less pronounced effect (Figure 7).

**FIGURE 7:**
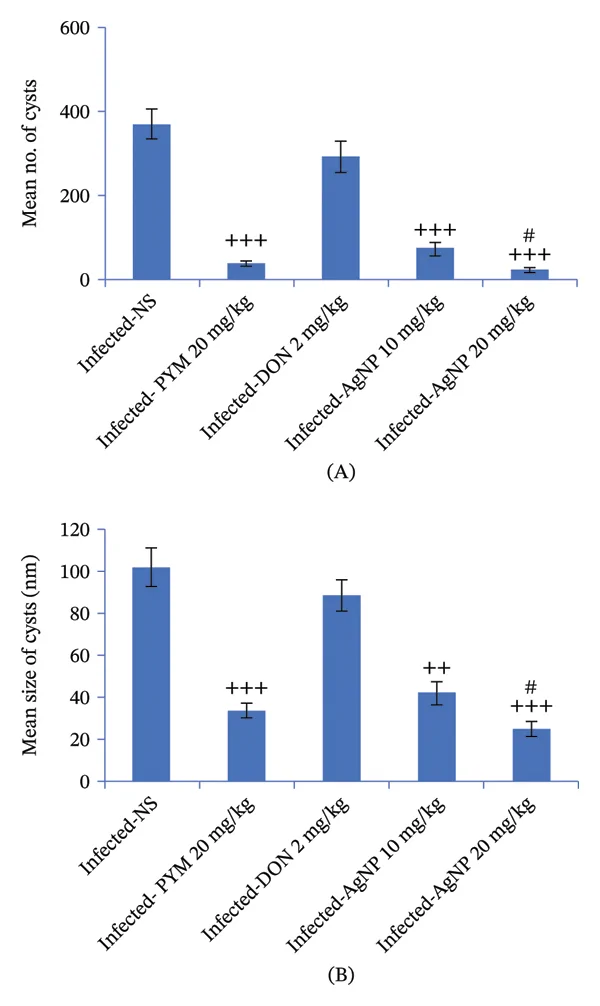
Evaluation of the impact of green‐synthesized silver nanoparticles (AgNP), donepezil (DON), and pyrimethamine (PYM) on the number (A) and dimensions (B) of tissue cysts in mice infected with *Toxoplasma gondii*, as assessed through microscopic examination. Data are expressed as mean ± standard deviation (*n* = 8 per group). Statistical significance is indicated as follows: ^++^ *p* < 0.01, and +++*p* < 0.001 relative to the *T. gondii*‐infected group; and ^#^ *p* < 0.05 when compared to the infected group treated with PYM. Post hoc comparisons were performed using Tukey’s honest significant difference (HSD) test following significant one‐way ANOVA results.

### 3.4. Evaluation of the Gene Expression Level

#### 3.4.1. *Bdnf* Gene

The infected untreated group exhibited a substantial decrease in *Bdnf* levels compared to the control group, with a 0.58‐fold reduction (*p* < 0.001). Administration of PYM resulted in a significant increase in *Bdnf* expression (1.57‐fold) relative to the infected group (*p* < 0.01); however, *Bdnf* levels in this group remained significantly lower than those observed following treatment with DON (2.79‐fold, *p* < 0.01) and AgNP at a dose of 20 mg/kg (3.59‐fold, *p* < 0.001). DON treatment significantly enhanced *Bdnf* expression compared to both PYM and AgNP at 10 mg/kg (*p* ranging from < 0.05 to < 0.01), although *Bdnf* levels were still significantly lower than those in the AgNP 20 mg/kg group (*p* < 0.01). Notably, AgNP induced a dose‐dependent increase in *Bdnf* expression, with the 20 mg/kg dose yielding significantly higher levels than the 10 mg/kg dose (*p* < 0.01) and all other treatment groups (*p* < 0.05) (Figure 8A).

**FIGURE 8:**
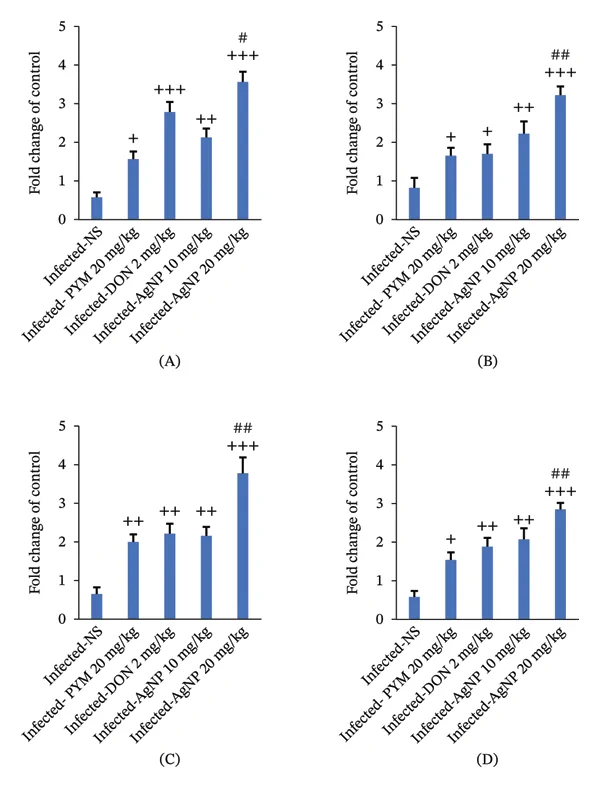
The effects of green‐synthesized silver nanoparticles (AgNP), donepezil (DON), and pyrimethamine (PYM) on the expression levels of (A) brain‐derived neurotrophic factor (*Bdnf*), (B) superoxide dismutase 2 (*Sod2*), (C) catalase (*Cat*), and (D) glutathione peroxidase 1 (*Gxp1*) in mice infected with *Toxoplasma gondii*, as assessed through real‐time PCR. Data are expressed as mean ± standard deviation (*n* = 8 per group). Statistical significance is indicated as follows: ^++^ *p* < 0.01, and ^+++^ *p* < 0.001 relative to the *T. gondii*‐infected group; and ^#^ *p* < 0.05 and ^##^ *p* < 0.01 when compared to the infected group treated with DON. Relative gene expression levels were normalized to *β*‐actin and calculated relative to the healthy noninfected control group using the 2^−ΔΔCt^ method. Post hoc comparisons were performed using Tukey’s honest significant difference (HSD) test following significant one‐way ANOVA results.

#### 3.4.2. *Sod2* Gene

Compared to the uninfected control group (set as 1.0‐fold), infection resulted in a significant reduction of *Sod2* expression to 0.83‐fold (*p* < 0.01), as illustrated in Figure 5B, where the control group serves as the reference baseline. Administration of PYM and DON each significantly elevated *Sod2* expression by approximately 1.7‐fold compared to the infected group (*p* < 0.01), with no statistically significant difference observed between these two treatments (*p* > 0.05). Treatment with AgNP at 10 mg/kg further increased *Sod2* expression was 2.24‐fold relative to drug‐treated groups (*p* < 0.01), while a higher dose of AgNP at 20 mg/kg induced a 3.24‐fold increase, which was significantly greater than all other groups (*p* < 0.05), indicating a pronounced dose‐dependent effect (Figure 8B).

#### 3.4.3. *Cat* Gene

*Cat* activity varied significantly among groups as determined by ANOVA (*p* < 0.001). Infection markedly decreased *Cat* levels to 0.69‐fold of control (*p* < 0.001). Both PYM (2.02‐fold) and DON (2.23‐fold) treatments significantly restored CAT activity compared to the infected group (*p* < 0.001), with no significant difference between these treatments (*p* > 0.05). AgNP at 10 mg/kg (2.19‐fold) elicited effects comparable to DON (*p* > 0.05), whereas the 20 mg/kg dose of AgNP (3.79‐fold) resulted in significantly higher *Cat* activity than all other treatments (*p* < 0.05) (Figure 8C).

#### 3.4.4. *Gpx1* Gene

*Gpx1* expression differed significantly across experimental groups (*p* < 0.001). Infection induced a substantial reduction in *Gpx1* to 0.59‐fold of control (*p* < 0.001). Treatment with PYM (1.56‐fold) and DON (1.91‐fold) significantly increased *Gpx1* expression relative to the infected group (*p* < 0.01 to *p* < 0.001), with DON demonstrating a significantly greater effect than PYM (*p* < 0.05). AgNP at 10 mg/kg (2.08‐fold) produced significantly higher *Gpx1* levels than PYM (*p* < 0.05) and were comparable to DON (*p* > 0.05). The 20 mg/kg AgNP dose (2.86‐fold) elicited the highest *Gpx1* expression, significantly exceeding all other groups (*p* < 0.05) (Figure 8D).

#### 3.4.5. *Nox2* Gene

Infection caused a dramatic increase in *Nox2* levels to 4.13‐fold relative to control (*p* < 0.001). PYM treatment reduced *Nox2* expression to 1.51‐fold compared to the infected group (*p* < 0.001) and resulted in lower levels than DON (2.06‐fold, *p* < 0.05). AgNP at 10 mg/kg (1.92‐fold) showed a moderate reduction in *Nox2*, not significantly different from DON (*p* > 0.05). Notably, AgNP at 20 mg/kg (1.23‐fold) produced the most pronounced suppression of *Nox2*, significantly lower than all other treatments (*p* < 0.05) (Figure 9A).

**FIGURE 9:**
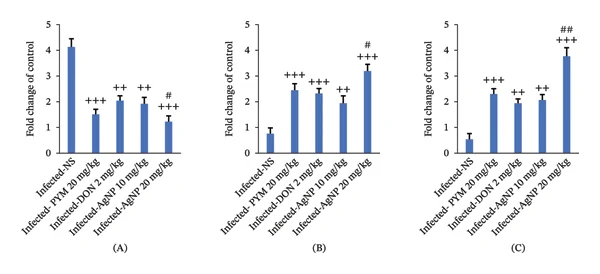
The effects of green‐synthesized silver nanoparticles (AgNP), donepezil (DON), and pyrimethamine (PYM) on the expression levels of (A) NADPH oxidase 2 (*Nox2*), (B) nuclear factor erythroid 2–related factor 2 (*Nrf2*), and (C) heme oxygenase‐1 (*Hmox1*) in mice infected with *Toxoplasma gondii*, as assessed through real‐time PCR. Data are expressed as mean ± standard deviation (*n* = 8 per group). Statistical significance is indicated as follows: ^++^ *p* < 0.01, and ^+++^ *p* < 0.001 relative to the *T. gondii*‐infected group; and ^#^ *p* < 0.05 and ^##^ *p* < 0.01 when compared to the infected group treated with DON. Relative gene expression levels were normalized to *β*‐actin and calculated relative to the healthy noninfected control group using the 2^−ΔΔCt^ method. Post hoc comparisons were performed using Tukey’s honest significant difference (HSD) test following significant one‐way ANOVA results.

#### 3.4.6. *Nrf2* Gene

*Nrf2* expression differed significantly among groups as confirmed by ANOVA (*p* < 0.001). Infection reduced *Nrf2* levels to 0.76‐fold of control (*p* < 0.01). Both PYM (2.46‐fold) and DON (2.34‐fold) significantly increased *Nrf2* expression compared to the infected group (*p* < 0.001), with no significant difference between these treatments. AgNP at 10 mg/kg (1.96‐fold) induced lower *Nrf2* expression than both drug treatments (*p* < 0.05). The 20 mg/kg AgNP dose (3.22‐fold) resulted in the highest *Nrf2* expression, significantly exceeding all other groups (*p* < 0.05) (Figure 9B).

#### 3.4.7. *Hmox1* Gene

*Toxoplasma* infection suppressed *Hmox1* to 0.57‐fold of control (*p* < 0.001). PYM treatment significantly increased *Hmox1* expression to 2.31‐fold relative to the infected group (*p* < 0.001) and was higher than DON treatment (1.96‐fold, *p* < 0.05). AgNP at 10 mg/kg (2.08‐fold) produced levels comparable to PYM (*p* > 0.05). The 20 mg/kg AgNP dose (3.77‐fold) elicited the greatest induction of *Hmox1*, significantly surpassing all other treatments (*p* < 0.05) (Figure 9C).

## 4. Discussion

This study elucidates the successful green synthesis of AgNP utilizing *M. neglectum* extract and demonstrates their neuroprotective effects against *T. gondii*‐induced cognitive impairment and explores the underlying Nrf2/BDNF and oxidative stress–related mechanisms. The initial confirmation of AgNP formation was achieved through the detection of a characteristic SPR band within the 400–450 nm range via UV–Vis spectroscopy. Consistent with established principles in nanomaterial characterization, this method primarily serves to verify nanoparticle formation rather than providing detailed structural information. The complementary use of SEM and DLS provided information regarding the morphology and size characteristics of the synthesized AgNPs. The difference between SEM‐derived particle dimensions and DLS measurements may reflect the distinction between dry‐state particle dimensions and the hydrodynamic behavior of particles in suspension [24, 25].

These physicochemical characteristics correspond closely with previously documented plant‐mediated AgNP, wherein biomolecules present in the extract function as both reducing and stabilizing agents, thereby modulating nanoparticle properties and biological activity [28]. Furthermore, FTIR spectroscopy confirmed the participation of functional groups such as phenolics and flavonoids in the synthesis and stabilization processes, corroborating prior findings regarding the critical role of phytochemicals in green nanotechnology applications [29].

Chronic *T. gondii* infection is increasingly recognized as a contributor to neurobehavioral and cognitive dysfunction, particularly deficits in spatial learning and memory. In the current investigation, untreated infected mice demonstrated pronounced impairments in the MWM task, characterized by extended escape latencies, increased travel distances, and diminished exploration of the target quadrant. These results align with prior research indicating that chronic toxoplasmosis disrupts hippocampal‐dependent cognitive functions [30, 31]. Our findings reveal that administration of green‐synthesized AgNP markedly enhanced spatial learning and short‐term memory in infected animals in a dose‐dependent manner. Mice treated with AgNP exhibited decreased escape latency and travel distance, alongside increased time spent in the target quadrant and higher crossing frequency, indicative of restored cognitive function. Notably, the higher dose of AgNP (20 mg/kg) elicited significantly greater improvements compared to the lower dose and other treatment groups. These findings suggest a neuroprotective potential of AgNP during chronic toxoplasmosis. Although research specifically addressing the impact of AgNP on cognitive dysfunction associated with toxoplasmosis remains limited, several studies have documented the neuroprotective potential of AgNPs in various neurological disorders [32, 33]. For instance, El‐Naggar et al. (2018) reported that biosynthesized AgNP significantly alleviated behavioral deficits and enhanced cognitive performance in rodents subjected to oxidative neural injury [34]. These findings are consistent with the behavioral improvements observed in the present study. The cognitive improvements observed following AgNP treatment are likely mediated through multiple complementary mechanisms. One plausible explanation is the significant reduction in parasite burden following AgNP treatment, which may mitigate parasite‐induced neuronal dysfunction and neuroinflammation. The chronic presence of *T. gondii* cysts in the brain has been shown to disrupt neurotransmitter homeostasis, alter synaptic connectivity, and impair neuronal communication, all of which contribute to cognitive decline [7]. Therefore, decreasing cyst number and size may directly enhance neuronal integrity and behavioral outcomes. Furthermore, AgNP may exert direct neuroprotective effects independent of their antiparasitic properties. Previous investigations suggest that green‐synthesized AgNP possess potent antioxidant and anti‐inflammatory activities capable of reducing neuronal oxidative damage and preserving hippocampal function [17]. AgNP may contribute to improved synaptic plasticity and neuronal survival, potentially through modulation of oxidative stress and neurotrophic signaling pathways. The superior efficacy of AgNP relative to DON observed in this study may thus reflect their broader therapeutic profile, encompassing both antiparasitic and neuroprotective actions.

The current investigation demonstrated that green‐synthesized AgNP exert a potent, dose‐dependent reduction in both the number and size of cysts during chronic *T. gondii* infection, exhibiting superior efficacy relative to PYM. These results are corroborated by an expanding corpus of literature underscoring the anti‐*Toxoplasma* properties of AgNP. Recent in vitro and in vivo research consistently indicates that biosynthesized AgNP significantly inhibits *T. gondii* proliferation. For example, Hematizadeh et al. reported that plant‐mediated AgNPs substantially decreased tachyzoite viability and parasite burden in infected models, with parasite counts markedly lower than those in untreated controls [35]. Similarly, Costa et al. [36] demonstrated that biogenic AgNP effectively controlled *T. gondii* infection in human trophoblast cells and placental explants by significantly reducing parasite invasion and intracellular replication. These findings align closely with our observation of a pronounced reduction in cyst burden following AgNP treatment. At the cellular level, several studies have elucidated mechanistic aspects of the antiparasitic activity of AgNPs. da Silva Sanfelice et al. found that AgNPs significantly diminished *T. gondii* infection and proliferation within macrophages, concomitant with increased production of ROS and proinflammatory mediators such as tumor necrosis factor‐alpha (TNF‐α) [37]. Earlier experimental work by Gaafar et al. also revealed that AgNPs significantly reduced parasite density in infected tissues and induced pronounced ultrastructural alterations in tachyzoites, including deformation, loss of motility, and structural disintegration [38]. The observed reduction in cyst size may suggest interference with cyst development; however, ultrastructural studies are required to confirm this hypothesis. Notably, whereas most prior studies have predominantly focused on acute infection models or tachyzoite stages, the present findings extend these observations to the chronic phase by demonstrating a substantial reduction in tissue cyst burden. This represents a critical advancement, given that tissue cysts constitute the principal therapeutic challenge in toxoplasmosis. The superior efficacy of AgNP, particularly at a dosage of 20 mg/kg, compared to PYM in reducing cyst numbers suggests that AgNP may target both tachyzoite replication and bradyzoite persistence. Discrepancies between the current results and some earlier reports may be attributable to variations in nanoparticle synthesis methods, physicochemical characteristics (including size, shape, and surface charge), and experimental models employed. It is well established that green‐synthesized nanoparticles often exhibit enhanced biological activity due to phytochemical capping agents, which may facilitate improved cellular uptake and bioavailability. Mechanistically, the pronounced antiparasitic effect observed herein is likely mediated through a combination of direct parasite toxicity and host‐directed modulation. AgNPs may directly disrupt parasite membranes, induce oxidative damage, and impair mitochondrial function, culminating in parasite death.

*T. gondii* infection is increasingly acknowledged as a contributor to neurocognitive impairments through mechanisms involving persistent neuroinflammation, oxidative stress, and disruption of neurotrophic signaling pathways [7]. In the current investigation, untreated infected mice demonstrated significant downregulation of *BDNF, Sod2, Cat, Gpx1, Nrf2,* and *Hmox1*, concomitant with marked upregulation of *Nox2*. These alterations confirm that toxoplasmosis induces substantial oxidative imbalance and compromises neuronal protective mechanisms. The results of the current investigation demonstrated that green‐synthesized AgNPs markedly enhanced learning and memory capabilities in mice infected with *T. gondii*. This improvement was accompanied by modulation of genes involved in the Nrf2‐associated antioxidant response, including *Nrf2, Hmox1, Sod2, Cat,* and *Gpx1*, together with increased *Bdnf* and reduced *Nox2* expression. These transcriptional changes are consistent with a potential role of redox and neurotrophic signaling in the observed effects; however, they do not establish direct activation or a causal role of the Nrf2/HO‐1–BDNF pathway. Furthermore, the observed significant upregulation of BDNF in the treated cohort suggests that the neuroprotective effects of AgNPs extend beyond the attenuation of oxidative stress, encompassing enhancements in synaptic plasticity and neuronal function. Recent literature supports that activation of the Nrf2/HO‐1 axis by AgNPs can mitigate neuroinflammation, decrease ROS, and promote neuronal survival and function [39, 40]. *T*. *gondii* infection is known to induce elevated ROS production, microglial activation, mitochondrial dysfunction, and hippocampal damage, culminating in deficits in learning and memory [7]. *NOX2*, a principal source of superoxide generation within the central nervous system, plays a critical role in exacerbating oxidative stress [8]. In this study, the observed downregulation of *Nox2* concomitant with increased expression of *Sod2, Cat, and Gpx1* suggests that AgNPs likely attenuate oxidative brain damage by suppressing ROS production and bolstering the endogenous antioxidant defense system. Additionally, the upregulation of *Hmox1* suggests enhanced transcriptional activity associated with the Nrf2/HO‐1 antioxidant response [39–41]. Although transcriptional modulation of antioxidant‐related genes was observed, direct biochemical assessment of oxidative stress biomarkers such as MDA, ROS, GSH, and antioxidant enzyme activity was not performed and should be addressed in future studies.

A further notable finding was the elevation of *Bdnf* expression following AgNP treatment. BDNF is a critical neurotrophin implicated in neurogenesis, neuronal survival, and synaptic plasticity, with diminished levels reported in various neurodegenerative and neuroinflammatory conditions [10]. Evidence indicates that oxidative stress and chronic inflammation suppress BDNF expression, whereas reduction of ROS and enhancement of mitochondrial function via Nrf2 activation create a conducive environment for increased BDNF expression and TrkB receptor‐mediated signaling [42]. Consequently, the cognitive improvements observed herein may be partially attributable to the interplay between reduced oxidative stress and augmented BDNF signaling. These findings align with previous studies. For instance, Sharma et al. (2023) demonstrated that biogenic AgNP reduced neuroinflammation and ROS production in human microglial cells (HMC3) through activation of the Nrf2/HO‐1 pathway [39]. Similarly, Gonzalez‐Carter et al. (2017) reported that AgNP mitigated neurotoxicity in dopaminergic neurons by decreasing neuroinflammation and oxidative stress [40]. Conversely, some investigations have indicated that high doses of AgNP can elevate ROS levels, inhibit Nrf2 activity, and induce oxidative damage, underscoring that the biological effects of AgNPs are contingent upon their dosage, size, and synthesis method [41, 43]. It appears that the green synthesis approach employed in the present study, which imparts plant‐derived phenolic and flavonoid compounds onto the nanoparticle surface, enhances biocompatibility and potentiates the antioxidant and neuroprotective properties of AgNP [19].

These findings suggest that AgNPs may exert therapeutic effects through both parasite‐related and host‐associated processes. AgNP treatment was associated with improved behavioral outcomes, reduced brain cyst burden, and modulation of selected oxidative stress‐ and neurotrophic‐related genes. However, as the present study did not include protein‐level analyses or pathway‐specific functional experiments, these findings should be interpreted as associations rather than direct evidence of a causal molecular mechanism. This study has several limitations. First, although the synthesized AgNPs were characterized using the available physicochemical methods, advanced techniques such as TEM, XRD, and elemental analysis were not performed. In addition, zeta‐potential analysis was not conducted; therefore, the surface charge and colloidal stability of the synthesized AgNPs could not be directly evaluated. Second, the molecular analyses were limited to gene expression measurements. Protein‐level validation of key markers, including NRF2, HMOX1, and BDNF, as well as antioxidant enzyme activity and pathway‐specific functional experiments, was not performed. Therefore, the observed transcriptional changes cannot establish activation or a causal role of the proposed molecular pathway. Third, direct markers of neuroinflammation, including inflammatory cytokines and glial activation, were not assessed. Therefore, the present findings should not be interpreted as direct evidence of attenuated neuroinflammation. In addition, molecular quantification of parasite burden and complementary behavioral tests were not performed. Finally, the long‐term safety, biodistribution, and potential toxicity of AgNPs remain to be investigated. Future studies incorporating comprehensive nanoparticle characterization, protein‐ and functional‐level validation, direct inflammatory assessments, and long‐term safety evaluation are needed to further clarify the therapeutic potential and underlying mechanisms of AgNPs.

## 5. Conclusion

In conclusion, green‐synthesized AgNP demonstrated promising antiparasitic and neuroprotective effects in experimental chronic toxoplasmosis, as evidenced by reduced brain cyst burden, improved cognitive performance, and modulation of oxidative stress‐ and neuroprotection‐related genes. It should be noted that the conclusions regarding the Nrf2/HO‐1–BDNF axis are based exclusively on RT‐qPCR gene expression data. Protein‐level validation through Western blotting and immunohistochemistry, as well as functional pathway assays, will be necessary to fully substantiate mechanistic claims and is planned for future investigations. In addition, further research is warranted to elucidate the precise molecular mechanisms involved, assess long‐term safety and toxicity, and confirm the therapeutic effectiveness of AgNP in expanded preclinical and clinical contexts.

## Author Contributions

Mojtaba Khaksarian and Hossein Mahmoudvand were responsible for the design and oversight of the experimental procedures. The experiments were performed, and data were collected by Amir Javadi Mamaghani, Erfan Babaeinejad, Pegah Shakib, and Saeedeh Shojaee. The initial draft of the manuscript was prepared by Hossein Mahmoudvand, Amal Khudair Khalaf, and Mojtaba Khaksarian.

## Funding

The authors have nothing to report.

## Disclosure

All authors have thoroughly reviewed and approved the final version of the manuscript for submission.

## Ethics Statement

All experimental procedures were conducted in accordance with internationally accepted guidelines for the care and use of laboratory animals (ARRIVE guidelines and NIH recommendations) and were approved by the Institutional Animal Ethics Committee of Lorestan University of Medical Sciences, Khorramabad, Iran (Approval No: IR.LUMS.REC.1398.021).

## Consent

The authors have nothing to report.

## Conflicts of Interest

The authors declare no conflicts of interest.

## Data Availability

The datasets used and/or analyzed during the current study are available from the corresponding author on reasonable request.
